# Discovery that PACAP, a mammalian neuropeptide, activates plant immunity through chemical screening

**DOI:** 10.3389/fpls.2026.1787727

**Published:** 2026-02-13

**Authors:** Guanghui Dong, Yingying Xu, Lijuan Zhou, Yajin Ye, Kunrong He

**Affiliations:** State Key Laboratory for Development and Utilization of Forest Food Resources, Co-Innovation Center for Sustainable Forestry in Southern China, Nanjing Forestry University, Nanjing, China

**Keywords:** disease resistance, neuropeptide, peptide, plant immune inducer, plant innate immunity (PTI)

## Abstract

Plants are constantly exposed to a variety of biotic stresses in their natural environment and rely on their immune systems to adapt to these challenges. Pattern-triggered immunity (PTI) and effector-triggered immunity (ETI) constitute two complementary layers of the plant innate immune system, both of which can be activated by immune elicitors. In this study, a *pFRK1-GUS* reporter system was employed to screen multiple natural product libraries, leading to the identification of the animal-derived neuropeptide pituitary adenylate cyclase-activating polypeptide (PACAP) and its truncated form, PACAP 6–38, as potential plant immune elicitors. Exogenous application of PACAP and PACAP 6–38 triggered multiple PTI-associated immune responses, including cytosolic calcium influx, MAPK phosphorylation, and induction of *FRK1* expression under the tested conditions, while notably failing to induce a detectable reactive oxygen species (ROS) burst. Moreover, pre-treatment with PACAP or PACAP 6–38 at the tested concentrations reduced bacterial titers of *Pseudomonas syringae* pv. *tomato* DC3000 by approximately 0.3–0.7 log units under single-application conditions. Notably, both peptides also enhanced plant resistance to *Ralstonia solanacearum*, indicating a broader role in bacterial disease resistance. Although the molecular receptors and downstream signaling components remain to be identified, this study establishes a proof-of-concept for cross-kingdom recognition of an animal neuropeptide by plants. Together, these findings highlight PACAP-induced immunity as being uncoupled from the canonical ROS burst, underscoring the conceptual novelty of animal-derived peptides as unconventional elicitors and providing a reference for potential future applications of PACAP in plants.

## 1 Introduction

Plants are fundamental to ecosystems, playing a crucial role in maintaining ecological stability. However, they frequently encounter threats from pathogenic microorganisms, such as bacteria, fungi, oomycetes, and viruses (Duxbury et al., 2021). Through long-term coevolution, plants have evolved an innate immune system that includes extracellular “sentinels” and intracellular “guards” to detect non-self molecules and initiate defense responses. This immune system consists of two interconnected layers: pathogen-associated molecular pattern (PAMP)-triggered immunity (PTI) and effector-triggered immunity (ETI) (DeFalco and Zipfel, 2021; Jones et al., 2016; Yu et al., 2017; Zhou and Zhang, 2020).

PTI serves as the first line of broad-spectrum defense, initiated by pattern-recognition receptors (PRRs) located on the cell membrane. These PRRs recognize various molecular patterns associated with pathogens, damage, microbes, or herbivores, known as PAMPs, damage-associated molecular patterns (DAMPs), microbe-associated molecular patterns (MAMPs), and herbivore-associated molecular patterns (HAMPs) (Ngou et al., 2022). This recognition activates downstream signaling pathways that help protect against most microbial colonization. However, some pathogens secrete effector proteins that can enter host cells, disrupting or suppressing PTI and enhancing their virulence. In response, plants were naturally selected for the production of intracellular nucleotide-binding leucine-rich repeat receptors (NLRs). These NLRs specifically detect effectors and activate the ETI defense (Jones and Dangl, 2006; Katagiri and Tsuda, 2010). Although the mechanisms for recognition and the intensity of the responses may differ, both PTI and ETI activate many shared downstream immune responses (Jones et al., 2024). Together, these coordinated reactions form a robust defense network that effectively limits pathogen invasion (Heath, 2000; Li et al., 2024; Ngou et al., 2021, Ngou et al., 2022).

In recent years, plant immune inducers have emerged as a promising strategy for sustainable crop protection due to their eco-friendly nature, low toxicity to mammals, broad-spectrum induction of resistance, and reduced risk of resistance development in pathogens (Yang et al., 2022). Among these, protein-based elicitors represent the earliest class to be commercialized and have been widely applied in agricultural production. A prominent example is the harpin protein-based formulation Messenger^®^, which is derived from *Erwinia amylovora* and is capable of activating multiple defense responses. This formulation has been extensively used in fruit and vegetable crops (Kulye et al., 2012). Similarly, the Hrip1 protein from *Alternaria tenuissima*, which is commercialized in China under the name “ATaiLing,” not only confers resistance against viral and fungal pathogens but also promotes plant growth and enhances yield and quality (Zhang et al., 2011). Another notable product is VDAL, derived from the fungus *Verticillium dahliae*. VDAL regulates plant hormone signaling pathways, activating both PTI and ETI simultaneously (Chun et al., 2024; Ma et al., 2021). Short peptide elicitors, such as Pep-13 from *Phytophthora sojae*, flg22 from bacterial flagellin, and nlp20 from necrosis- and ethylene-inducing peptide 1-like proteins (NLPs), are also widely recognized by plant receptors and can effectively trigger immune responses in both host and non-host plants. Overall, protein- and peptide-based elicitors constitute a well-established category of plant immune inducers, presenting significant potential for reducing pesticide use and advancing sustainable agriculture.

Pituitary adenylate cyclase-activating polypeptide (PACAP) was originally isolated from bovine pituitary tissue and is classified as a member of the vasoactive intestinal peptide family (Vaudry et al., 2000). As a highly conserved neuropeptide, PACAP is widely distributed in the central and peripheral nervous systems. It regulates a wide range of physiological processes, such as circadian rhythms, reproductive development, cognitive function, pain perception, neuroprotection, and neuromodulation (Li et al., 2025; Chen et al., 2025; Cherait et al., 2020, Cherait et al., 2025; Pataki et al., 2002). PACAP functions primarily in two biologically active forms: the full-length 38-amino acid peptide known as PACAP 1–38 and a C-terminally truncated variant called PACAP 1-27. Of these, PACAP 1–38 is the predominant isoform in the nervous system, accounting for approximately 90% of the total peptide content (Miyata et al., 1989, Miyata et al., 1990). Additionally, a synthetic peptide derivative, PACAP 6-38, functions as a potent antagonist of PACAP receptors by selectively blocking receptor-mediated signaling pathways. This derivative has been widely used in research examining neuroprotection, ischemic brain injury, apoptosis, and tumor formation, making it a useful tool for understanding the physiological and pathological roles of PACAP signaling (Farnham et al., 2011; Leyton et al., 1998; Lin et al., 2011).

In this study, a reporter-based high-throughput screening system was utilized to identify novel compounds that activate plant immunity. Through systematic screening of several chemical and peptide libraries, PACAP and PACAP 6–38, were identified as plant immune elicitors. Exogenous application of both peptides induced cytosolic Ca²^+^ influx and MAPK activation in *Arabidopsis thaliana*. These results broaden the functional context in which PACAP peptides can be studied and provide additional evidence that PACAP is capable of activating plant immune signaling.

## 2 Materials and methods

### 2.1 Chemicals

Chemical libraries were purchased from TargetMol (Shanghai, China) and Selleck (Houston, TX). Synthetic PACAP 1-38, PACAP 1-27, and PACAP 6-38 (≥95% purity) were obtained from GenScript (Nanjing, China) and dissolved in sterile water. The identity and purity of the synthesized PACAP peptides were confirmed by mass spectrometry (,**Supplementary Figure S4**).

### 2.2 Plant materials and growth conditions

Wild-type Arabidopsis ecotype Col-0, corresponding transgenic lines, and *Nicotiana benthamiana* were used in this study. Unless otherwise specified, all plants were cultivated in a controlled-environment greenhouse. Growth conditions were maintained at 20–25 °C under a 16-h light/8-h dark photoperiod.

For Arabidopsis and *N. benthamiana*, seeds were surface sterilized by immersion in 30% (v/v) sodium hypochlorite solution containing 0.02% (v/v) Tween-20 for 15 min with gentle agitation, followed by five rinses with sterile distilled water to remove residual sterilizing agents. The sterilized seeds were subjected to cold stratification at 4 °C for 2–3 days to break dormancy and promote synchronized germination. Seeds were then sown on 1/2 Murashige and Skoog (MS) (PhytoTechLABS, Beijing, China) basal medium (pH 5.7) supplemented with 0.5% (w/v) sucrose as a carbon source and solidified with 0.4% (w/v) Phytagel (OriLeaf, Nanjing, China). Plates were placed vertically in a controlled growth chamber under continuous white light (40 μmol·m⁻²·s⁻¹) at 22 °C for seedling cultivation. After 7–10 days, seedlings were either transferred to soil or directly used for subsequent experiments, depending on the experimental design.

### 2.3 GUS staining

For histochemical staining of transgenic lines, the predicted *FRK1* promoter (~2 kb upstream of the ATG start codon) was amplified from Col-0 genomic DNA. The *proFRK1::GUS* construct was cloned into the pGWB3 vector and then introduced into Col-0 plants by the floral dip method. Homozygous T3 plants were selected for analysis. Seedlings were vacuum infiltrated with staining solution containing 100 mM NaH_2_PO_4_ (pH 7.0), 10 mM Na_2_EDTA, 0.5 mM K_4_[Fe(CN)_6_]·3H_2_O, 0.5 mM K_3_;[Fe(CN)_6_], 0.1% (v/v) triton X-100, and 1 mM X-Gluc (5-bromo-4-chloro-3-indolyl β-D-glucuronide, cyclohexylammonium salt). Samples were incubated at 37 °C in the dark for 14 h. Chlorophyll was removed with 95% ethanol until tissues were clear, and staining patterns were documented by photography. The histochemical assay of GUS activity was carried out as previously reported (Ye et al., 2020).

### 2.4 RNA Extraction and reverse transcribed quantitative PCR RT-qPCR

Total RNA was extracted from plant tissues using the Ultrapure RNA Kit (Cowin Biotech, China). RNA integrity was verified by agarose gel electrophoresis, and concentration and purity were measured using a NanoDrop spectrophotometer (Thermo Fisher Scientific, Nanjing, China). One microgram of total RNA was treated with genomic DNA wiper and reverse-transcribed into cDNA using the HisScript II Q RT SuperMix kit (Vazyme, Nanjing, China). RT-qPCR was performed with ChamQ Universal SYBR qPCR Master Mix (Takara, Beijing, China) on a Bio-Rad CFX Connect™ Real-Time PCR Detection System. Relative expression levels were calculated using the 2^−ΔΔCt^ method, with *EF1a* as an internal control. Each assay included three biological replicates and three technical replicates.

### 2.5 MAPK activity assay

Seven-day-old Arabidopsis seedlings grown on 1/2 MS medium under growth conditions maintained at 20–25 °C with a 16-h light/8-h dark photoperiod were transferred into 24-well plates containing sterile water one day before treatment and incubated overnight (approximately 8 h). On the following day, seedlings were treated with PACAP peptide solution for the indicated times (e.g., 15 min, 30 min, and 1 h). Frozen tissues were ground with 3-mm zirconia beads using a FastPrep 24 grinder, and total proteins were extracted in buffer containing 50 mM Tris–HCl (pH 7.5), 150 mM NaCl, 10% glycerol, 2 mM EDTA, 5 mM DTT, 1× EDTA-free protease inhibitor cocktail (Roche), and 1% Triton X-100. Equal amounts of protein (14 μL per lane) were subjected to SDS–PAGE using 4–20% gradient gels and transferred to PVDF membranes (Millipore) at 100 V for 1 h.

Phosphorylated MPK3 and MPK6 were detected using rabbit monoclonal anti-phospho-p44/42 MAPK (Erk1/2) antibody (Cell Signaling Technology, Shanghai, China; 1:2000). HRP-conjugated goat anti-rabbit IgG (Abmart, Shanghai, China; 1:12,000) was used as the secondary antibody. Blots were visualized by ECL and imaged with a Tanon 3600 system (Tanon, Beijing, China), with exposure times of 30 s–2 min. All experiments were repeated at least three times with consistent results.

### 2.6 Calcium influx assays and imaging

Calcium influx was measured using the Col-Q (Columbia aequorin) Arabidopsis line as previously reported (Choi et al., 2014). A 100 μL mixture containing CaCl_2_ (1 M, 100×), MES (200 mM, 100×), and coelenterazine (CTZ, 1 mM, 100×) was prepared and added to each well of a 96-well plate. Seven-day-old seedlings grown on 1/2 MS medium were transferred into the plate and incubated overnight (approximately 8 h) to allow aequorin reconstitution.

On the following day, 50 μL of treatment solutions were added to each well. PACAP and PACAP 6–38 were dissolved in sterile water and applied at final concentrations of 20, 50, and 100 μM. Luminescence signals were recorded for 30 min using a High Sensitivity Plate Luminescence Detector (BLT Lux-P110, Guangzhou, China), and peak luminescence values were used for quantification. Each data point was measured using 8 seedlings (technical replicates), and the experiment was independently repeated three times, yielding consistent results.

Imaging was performed using a Tanon 3600 imaging system (Tanon, Beijing, China) to quantify calcium influx, with 10–15 seedlings per well. The experiment was repeated three times.

### 2.7 Bacterial infection assay

*P. syringae* pv. *tomato* DC3000 was cultured on King’s B (KB) agar medium (12.5 g/L King’s B base, 15 g/L agar, 10 mL/L glycerol) at 28 °C for 48 h. Single colonies were inoculated into 4 mL of KB liquid medium and grown overnight at 28 °C with shaking at 200 rpm until the OD_600_ reached 0.6–0.8. The bacterial cells were harvested by centrifugation and resuspended in 10 mM MgCl_2_ to an OD_600_ of 0.002 for inoculation.

To examine whether peptide pretreatment enhances plant resistance, fully expanded leaves of 4-week-old Arabidopsis plants were infiltrated with PACAP or its antagonist PACAP 6–38 one day before bacterial inoculation using a 1 mL needleless syringe. Control plants were treated with sterile water (mock). After 24 h, leaves were inoculated with *P. syringae* pv. *tomato* DC3000 suspension as described above.

After infiltration, excess bacterial solution was removed with sterile filter paper, and plants were maintained under high-humidity conditions in a growth chamber. At 2 or 3 days post-infection (dpi), three leaf discs (0.5–0.6 cm diameter) per leaf were collected, surface-sterilized with 75% ethanol for 30 s, rinsed twice with sterile distilled water, and homogenized in 10 mM MgCl_2_. Serial dilutions of the homogenates were plated on KB agar containing rifampicin (50 μg/mL). Plates were incubated at 28 °C for 48 h before counting colony-forming units (CFUs). For each biological replicate, at least nine individual plants were analyzed, and the experiment was repeated three times with consistent results.

For the *R. solanacearum* soaking infection assay, two-week-old *Nicotiana tabacum* cv. Yabuli seedlings grown on 1/2 MS agar medium plates were gently removed and recovered in sterile ddH_2_O overnight prior to inoculation. Roots were slightly wounded using a sterile blade to facilitate bacterial entry. Seedlings were then infiltrated with PACAP, PACAP 6–38, or ddH_2_O as a mock control. Twelve hours after peptide or mock treatment, a suspension of *R. solanacearum* at a concentration of 1 × 10^6^ CFU mL⁻¹ was infiltrated into the same tissues.

Samples were collected at 1 day post-inoculation for bacterial quantification. Tissues were weighed, homogenized in sterile water, and serial dilutions were plated onto solid CPG medium. Colony-forming units (CFUs) were counted after incubation at 28 °C for 2 days and expressed as CFU per gram of fresh tissue.

### 2.8 ROS production measurement

Leaf discs (0.125 cm²) were excised from the 7th to 9th true leaves of 4-week-old Arabidopsis plants and placed in 96-well plates containing 100 µL sterile water. Samples were incubated overnight under low light conditions. The water was then replaced with 100 µL of reaction solution containing 20 µM L-012 (TargetMol Chemicals Inc., Shanghai, China), 10 µg/mL horseradish peroxidase (Beyotime Biotechnology, Shanghai, China), and either flg22 or the test chemicals. Chemiluminescence was recorded immediately for 40–90 min using a High Sensitivity Plate Luminescence Detector (BLT Lux-P110). The experiment was repeated three times with consistent results.

## 3 Results

### 3.1 PACAP induces FRK1 expression as an immune elicitor

**Figure 1:**
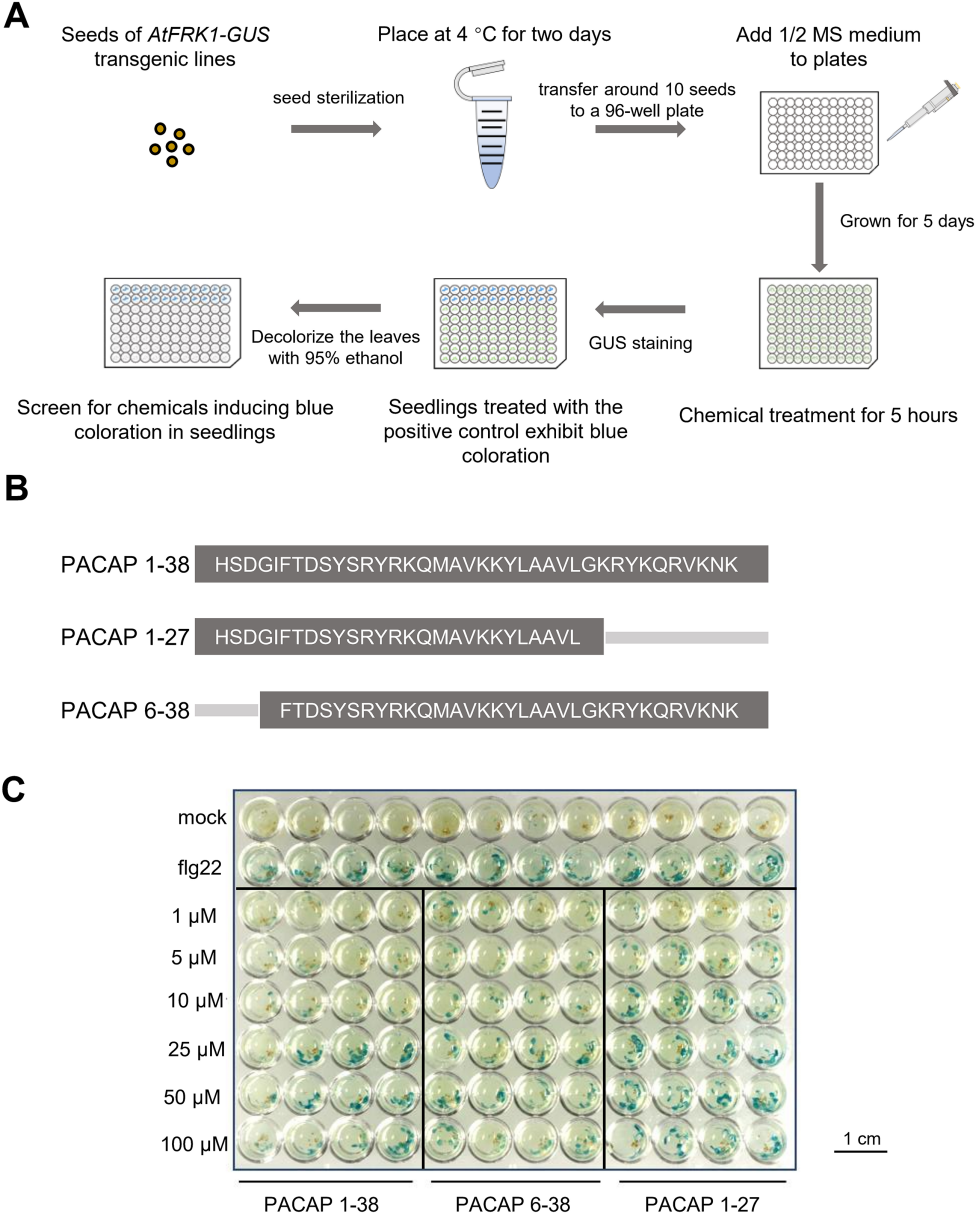
Neuropeptide PACAP activates *FRK1* expression. **(A)** Schematic workflow of the chemical screen for inducers of *pFRK1-GUS* expression. Individual chemicals were added to wells of a 96-well plate containing 5-day-old *pFRK1-GUS* seedlings and incubated for 5 h, followed by histochemical GUS staining. **(B)** Amino acid sequences of the peptides PACAP and its truncated variants. **(C)** PACAP activates *pFRK1-GUS* expression in a concentration-dependent manner. Transgenic *pFRK1-GUS* seedlings grown for 5 days on 1/2 MS medium were soaked in the indicated concentrations of PACAP. After 5 h of treatment, GUS staining was performed to detect *FRK1* expression. Treatment with 100 nM flg22 served as a positive control, and sterile water served as the negative control. *p < 0.05.

*FLG22-INDUCED RECEPTOR-LIKE KINASE 1* (*FRK1*) is an early marker gene of PTI in Arabidopsis (Chinchilla et al., 2007; Thor et al., 2020). In this study, a *FRK1* promoter-based high-throughput screening system was employed using transgenic *pFRK1-GUS* seedlings, in which the *FRK1* promoter is fused to the β-glucuronidase (GUS) reporter gene (**Figure 1A**). A large-scale screening was conducted using *pFRK1-GUS* Arabidopsis reporter seedlings, which were exposed to approximately 10,000 compounds from various commercial libraries. Five-day-old seedlings were grown under uniform conditions in 96-well plates and treated with individual compounds, and GUS staining was evaluated 24 hours after treatment. Primary hits were selected based on visibly intensified blue coloration compared to the negative control (**Figure 1A**).

Since GUS staining served only as a preliminary indicator of immune activation, a subset of these candidates underwent secondary validation through immune response assays, including MAPK phosphorylation, defense gene expression, and ROS production, to eliminate false positives and confirm genuine immune activation (Ma et al., 2025). Following this validation, it was discovered that the neuropeptide PACAP and its receptor antagonist fragment PACAP 6–38 function as plant immune elicitors. Notably, both PACAP 1–27 and PACAP 6–38 are truncated derivatives of PACAP 1-38 (**Figure 1B**). All three PACAP peptides activated the expression of *pFRK1-GUS* in a concentration-dependent manner (**Figure 1C**; ,**Supplementary Figure S1**). Subsequent experiments employed 20 μM PACAP based on these dose-response observations, as lower concentrations produced minimal or inconsistent immune responses. Together, these results indicate that PACAP may play a role in plant defense responses by activating early immune gene expression.

### 3.2 PACAP triggers typical plant immune responses

**Figure 2:**
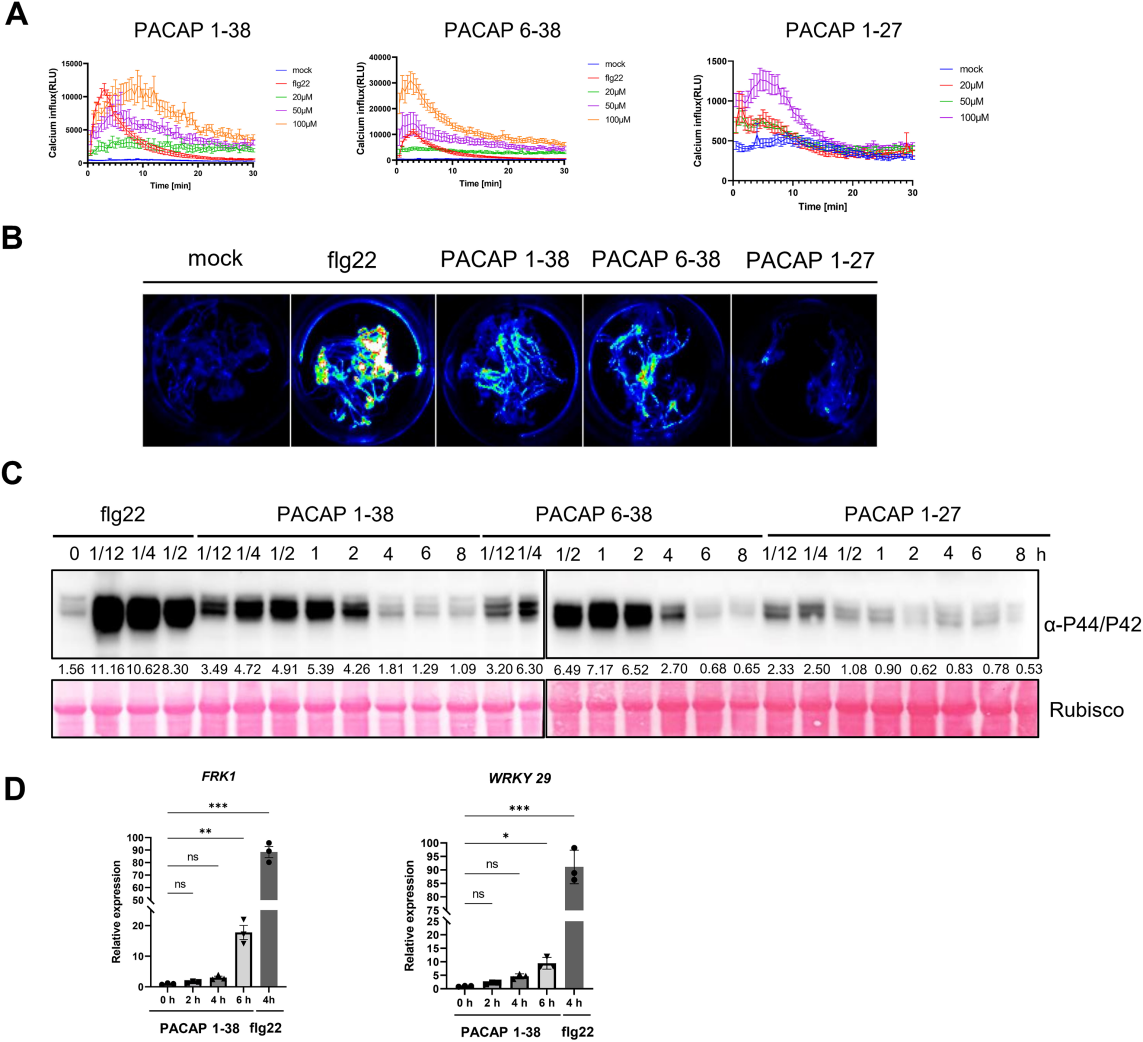
PACAP functions as an elicitor to trigger plant immune responses. **(A)** Quantification of calcium influx in Col-Q seedlings expressing aequorin. Luminescence signals were recorded for a period of 30 min following treatment of Col-Q seedlings with PACAP 1-38, PACAP 6-38, or PACAP 1–27 at concentrations of 20, 50, and 100 μM, as indicated. Seven-day-old seedlings were preloaded overnight with coelenterazine before measurement. Values are means ± SEM (n = 8 biological replicates). Col-Q: Col-aequorin. **(B)** Calcium ion influx was measured in aequorin transgenic seedlings for 30 min after treatment with mock (sterile water), 100 nM flg22, 50 μM PACAP 1-38, 50 μM PACAP 6-38, or 50 μM PACAP 1-27. **(C)** PACAP induces MAPK activation. Arabidopsis seedlings were soaked in 50 μM PACAP 1-38, 50 μM PACAP 6-38, or 50 μM PACAP 1–27 for the indicated times, followed by immunoblotting with an anti-p44/p42 antibody. 100 nM flg22 served as a positive control. Ponceau S staining served as the loading control. **(D)** PACAP 1–38 induces the expression of defense-related marker genes *FRK1* and *WRKY29*. Col-0 seedlings were soaked in 25 μM PACAP 1–38 for 0, 2, 4, and 6 h before analysis of *FRK1* and *WRKY29* transcript levels by RT-qPCR. Flg22 treatment served as a positive control. Values are means ± SEM (n = 3 biological replicates). *p < 0.05, **p < 0.01, ***p < 0.001(two-tailed Student’s *t* test).

Calcium serves as a crucial second messenger in all eukaryotic cells and plays a vital role in plant immune signaling (Jiang and Ding, 2023). Under normal physiological conditions, the concentration of free calcium in the apoplast is significantly higher than in the cytosol (Demidchik et al., 2018). Upon pathogen infection or elicitor recognition, a rapid calcium influx is triggered, which initiates downstream immune signaling (Zipfel and Oldroyd, 2017; Yuan et al., 2017). To investigate the effects of PACAP on the induction of free cytosolic calcium ([Ca^2+^]_cyt_), an aequorin-based Ca^2+^ reporter system combined with a luminescence microplate reader was used. The quantitative analysis showed that PACAP 1–38 rapidly induced [Ca^2+^]_cyt_ in Arabidopsis in a dose-dependent manner. Notably, both PACAP 1–38 and PACAP 6–38 induced significant calcium signaling, while PACAP 1–27 exhibited relatively weak activity compared to the other two variants (**Figure 2A**). Additionally, the *in vivo* bioluminescence imaging results further confirmed the increase in [Ca^2+^]_cyt_ after PACAP 1–38 and PACAP 6–38 treatments (**Figure 2B**).

To explore whether PACAP and its truncated form, PACAP 6-38, could activate other hallmark events in plant immunity, the activation of MAPK was examined by immunoblot analysis. The results demonstrated that treatment with either PACAP 1–38 or PACAP 6–38 effectively induced MAPK phosphorylation in Arabidopsis seedlings (**Figure 2C**). And this induction was detectable as early as 15 minutes after treatments, which mirrored PAMP-induced MAPK phosphorylation (**Figure 2C**). RT-qPCR analysis indicated that PACAP-induced *FRK1* expression, which was consistent with the *pFRK1-GUS* results (**Figure 2D**). Furthermore, the expression of *WRKY29*, another immune marker gene, was induced by PACAP1–38 in a time-dependent manner (**Figure 2D**). Interestingly, although PACAP triggered Ca²^+^ influx, MAPK phosphorylation, and defense gene expression, none of the three variants (PACAP 1–38, 1–27, 6–38) induced a detectable ROS burst at 20–100 μM over two hours. Control assays with flg22 confirmed the assay’s sensitivity, indicating that PACAP activates immune responses without a canonical ROS burst (,**Supplementary Figure S2**).

### 3.3 PACAP enhances plant resistance to bacterial pathogens

**Figure 3:**
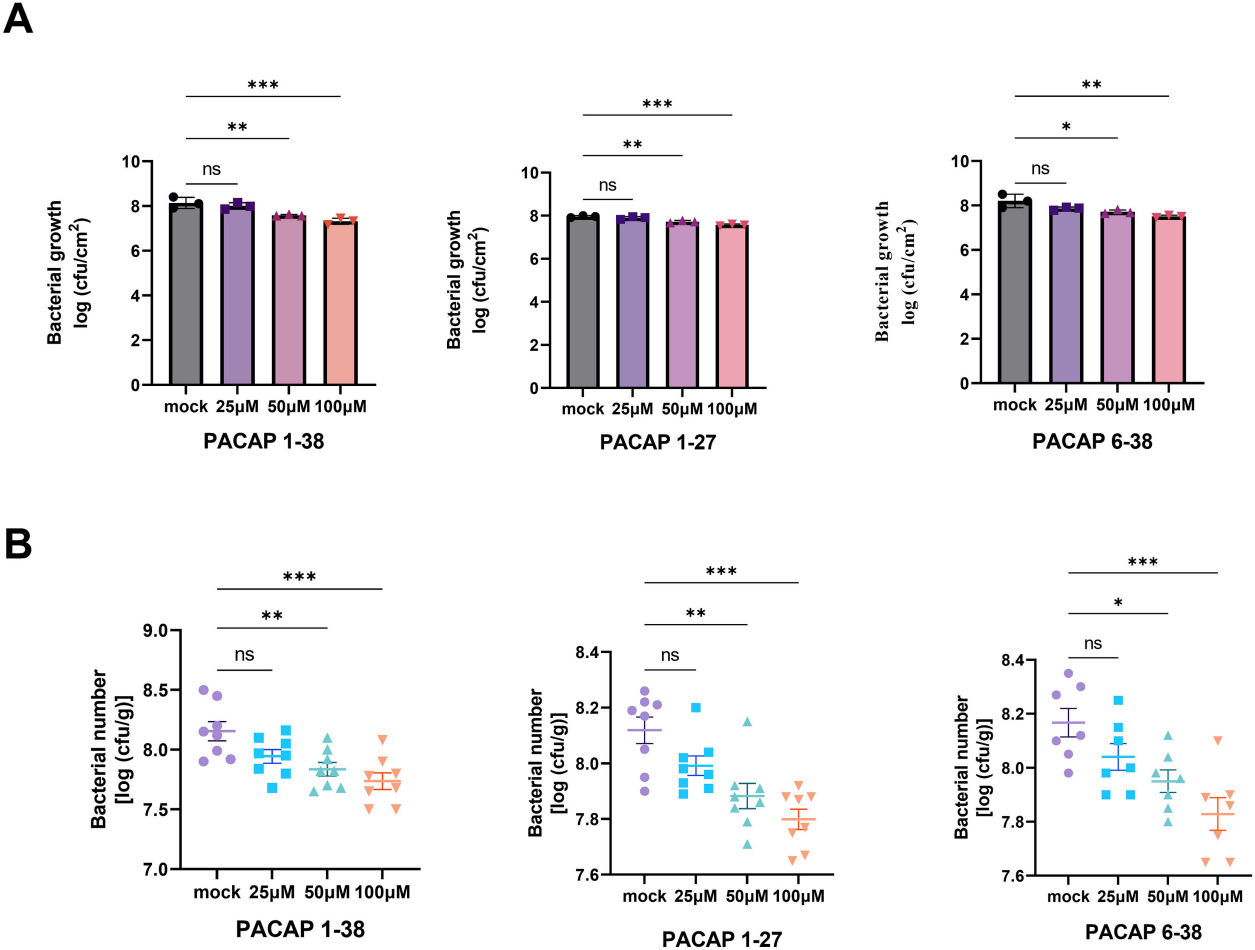
PACAP peptides promote bacterial disease resistance in plants. **(A)** Exogenous application of PACAP 1-38 、PACAP 1–27 or PACAP 6–38 enhances resistance of Arabidopsis to *P. syringae* pv. *tomato* DC3000. Col-0 plants were pretreated with different concentrations (25 μM, 50 μM, and 100 μM) PACAP 1–38 for 24 h prior to *P. syringae* pv. *tomato* DC3000 inoculation. Bacterial populations were quantified 2 days post-inoculation. Values are means ± SEM (n = 3 biological replicates). ns, not significant, *p < 0.05, **p < 0.01, ***p < 0.001(two-tailed Student’s *t* test). **(B)** PACAP 1-38 、PACAP 1–27 or PACAP 6–38 restricts the growth of *R. solanacearum* in *Nicotiana tabacum* cv. *Yabuli*. Seedlings were pretreated with sterile water 、PACAP 1-38 、PACAP 1–27 or PACAP 6–38 for 12 h, followed by syringe infiltration with a 10^6^ cfu/mL bacterial suspension. Bacterial growth was determined 48 h after inoculation. Values represent means ± SEM (n = 8 plants per strain). ns, not significant, *p < 0.05, **p < 0.01, ***p < 0.001(two-tailed Student’s *t* test).

Next, it was examined whether PACAP-triggered early immune responses are sufficient to confer effective pathogen resistance in plants. Following inoculation with pathogenic strain *P. syringae* pv. *tomato* DC3000, the results indicated that both PACAP 1–38 and PACAP 6–38 enhanced plant resistance to this pathogen (**Figure 3A**). Consistently, PACAP 1-38, PACAP 1–27 and PACAP 6–38 also enhanced plant resistance to *R. solanacearum* (**Figure 3B**). Additionally, *in vitro* growth assays were conducted to evaluate the antimicrobial activity of PACAP, which revealed that PACAP did not directly inhibit the growth of the tested pathogens (,**Supplementary Figures S3A, B**). In summary, through the chemical screening and functional validation, it was identified that PACAP is an effective plant immunity inducer that activates several hallmark responses associated with early immune signaling.

## 4 Discussion

This study identified PACAP and its derivative PACAP 6–38 as animal-derived peptides capable of activating multiple immune responses in Arabidopsis, including cytosolic calcium influx, MAPK activation, and defense gene expression. Notably, PACAP did not elicit a detectable ROS burst. This divergence from a canonical PTI profile—similar to what has been reported for other receptor-mediated pathways, such as that triggered by the quinone DMBQ (Laohavisit et al., 2020) or by metformin, which also induces MAPK activation and defense gene expression without a ROS burst—suggests that PACAP activates only a subset of PTI-associated signaling components rather than a full canonical PTI program. Such selective activation implies that immune signaling outputs can be differentially engaged, potentially allowing enhanced defense responses while limiting excessive oxidative stress. Together, these findings support a role for PACAP as an effective immune signaling molecule in plants.

In contrast to previously characterized plant immune elicitors (Derevnina et al., 2016; Montesano et al., 2003; Schwessinger and Ronald, 2012; Boutrot and Zipfel, 2017). PACAP is distinctive in that it originates from an animal source. This cross-kingdom activity suggests a modest conceptual novelty compared with classical plant immune elicitors, which are typically derived from microbes or plants themselves. The rapid and transient MAPK activation triggered by exogenous PACAP application suggests that its perception likely occurs at or near the cell surface. In animals, PACAP is recognized by G protein-coupled receptors (PAC1-R and VPAC1/2), which mediate neuronal and immune signaling (Jansen-Olesen and Hougaard Pedersen, 2018). Although plants possess heterotrimeric G proteins, the existence of bona fide GPCRs in plants remains controversial. Instead, plant G proteins are often coupled to single-pass receptor-like kinases (RLKs) or receptor-like proteins (RLPs) which serve as primary sensors for extracellular ligands (Pandey, 2019; Stateczny et al., 2016).

Among potential receptor candidates involved in PACAP perception, several immune-related receptor families warrant consideration. The leucine-rich repeat receptor-like kinase (LRR-RLK) family—including well-characterized elicitor receptors such as FLAGELLIN-SENSING 2 (FLS2), ELONGATION FACTOR Tu RECEPTOR (EFR), and PLANT ELICITOR PEPTIDE RECEPTORS (PEPRs)—represents the most plausible class, given their established roles in peptide and PAMP recognition (Zipfel, 2014). In addition, receptor-like proteins that signal through SUPPRESSOR OF BIR1-1 (SOBIR1)–BRI1-ASSOCIATED RECEPTOR KINASE 1 (BAK1) complexes, as well as wall-associated kinases (WAKs) and malectin-like RLKs, could also participate in PACAP detection, as these receptor classes are known to recognize structurally diverse extracellular ligands (Albert et al., 2015; Kohorn, 2016).

To further explore whether PACAP perception depends on known immune signaling components, we examined PACAP-induced MAPK activation in a series of *Arabidopsis* immune-related mutants, including *lore*, *hpcal1*, *lyk4/lyk5*, *fls2 efr cerk1* (fec), and *bak1 bkk1 cerk1* (bbc). Notably, PACAP 1–38–induced MAPK phosphorylation was not markedly compromised in any of these mutant backgrounds under the tested conditions. In addition, Sequence comparison analyses revealed that PACAP does not share significant homology or conserved motifs with known plant peptides, suggesting that its recognition in plants is unlikely to result from homology-based molecular mimicry. While current data support extracellular perception leading to early immune activation, direct evidence for specific receptor involvement is still lacking. Identifying the receptor(s) responsible for PACAP perception and delineating the downstream signaling pathway will therefore be a key priority for future research.

Overall, this work demonstrates that animal-derived peptides such as PACAP can be perceived by plants and activate immune-related signaling pathways. Rather than providing a complete mechanistic model, our findings offer a reference for the existence of unconventional, cross-kingdom immune elicitors and highlight their potential relevance to plant immunity. This study thus broadens the conceptual landscape of immune elicitor research while leaving detailed mechanistic dissection to future investigation.

## Data Availability

The raw data supporting the conclusions of this article will be made available by the authors, without undue reservation.
