# Supplementary material for: Discovery that PACAP, a mammalian neuropeptide, activates plant immunity through chemical screening
Source: Front Plant Sci. 2026 Feb 13;17:1787727. doi: 10.3389/fpls.2026.1787727 (PMC12946033; doi:10.3389/fpls.2026.1787727)
Supplement: Supplementary file 1 [file DataSheet1.pdf]

Supplementary Figure 1

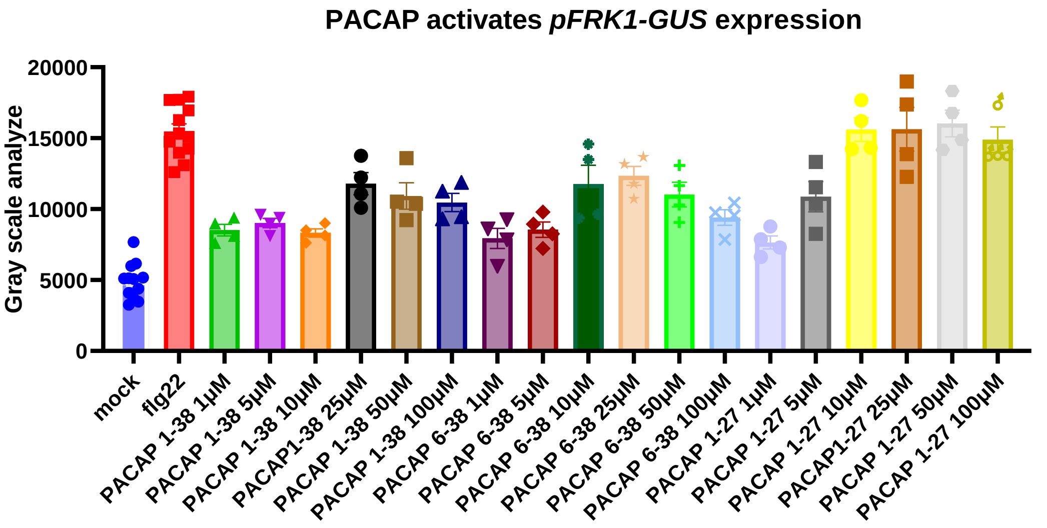

Supplementary Figure 1. Quantification of the staining intensity in Figure 1C.

Supplementary Figure 2

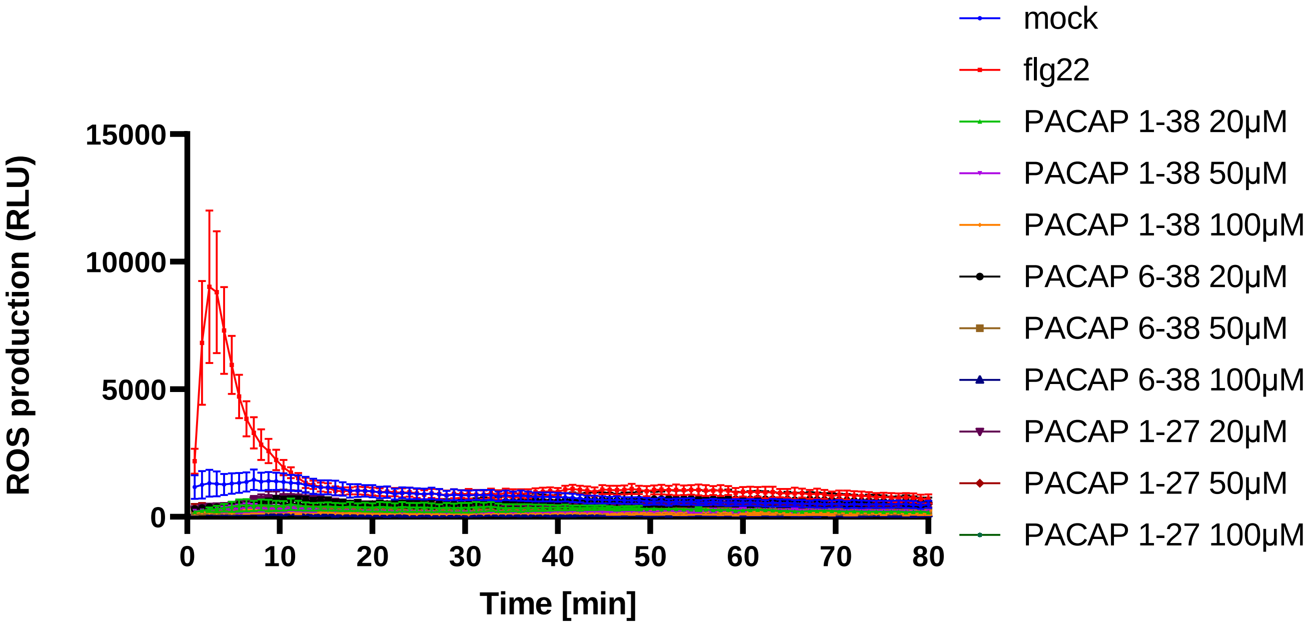

Supplementary Figure 2. Quantification of the production of ROS by PACAP and PACAP 6-38 in Arabidopsis. Col-0 leaf discs were treated with different concentrations of chemical compounds for 30 minutes. Values are means ± SEM (n = 8 biological replicates).

Supplementary Figure 3

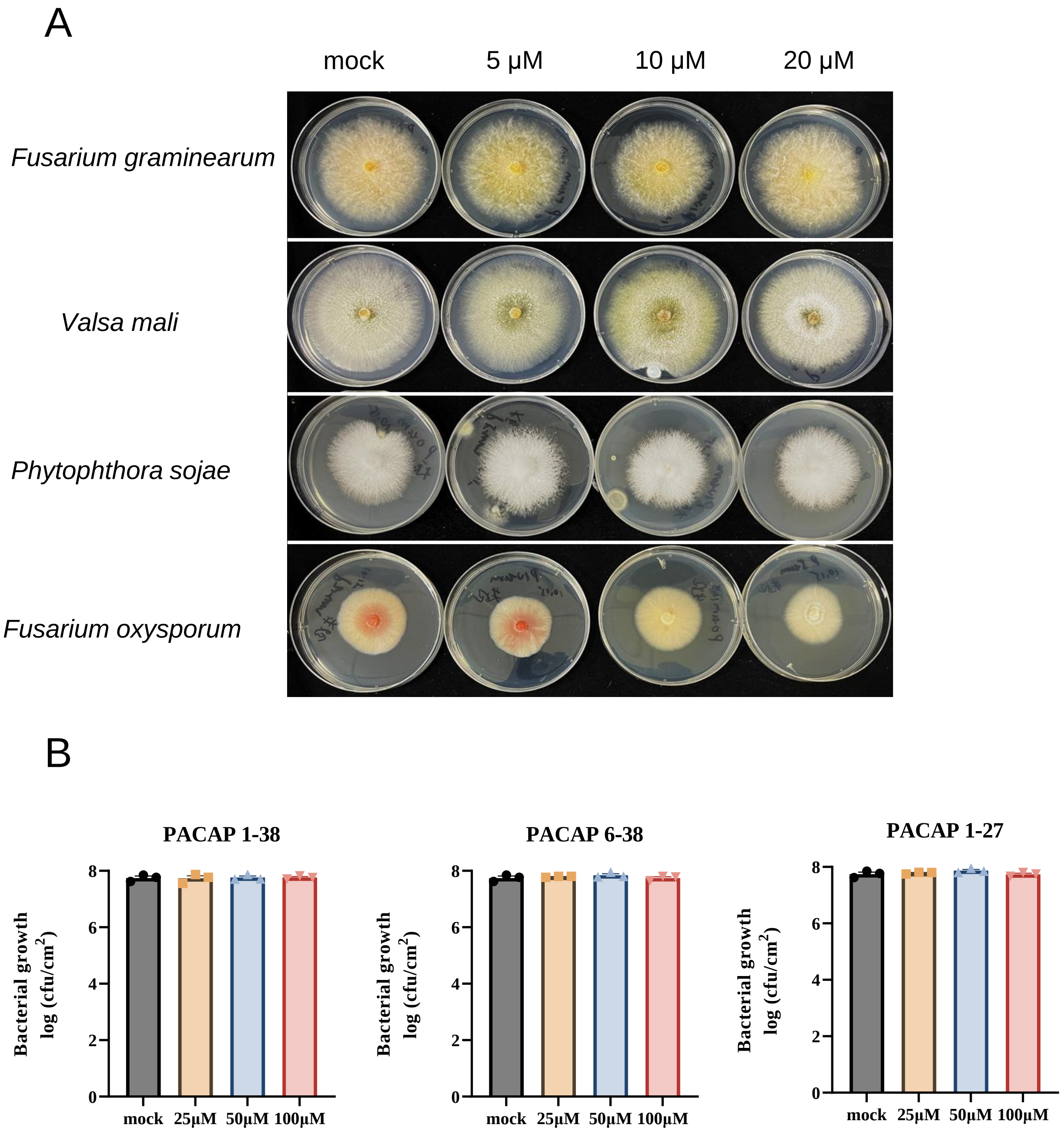

Supplementary Figure 3. The sensitivity of phytopathogens to PACAP. (A). In all these *in vitro* growth assays, mycelial plugs measuring 3 mm in diameter were inoculated onto medium containing the indicated concentrations of PACAP 1-38. (B). Bacterial suspensions of *P. syringae* pv. *tomato* DC3000 (1 × 10^7 CFU mL^{-1}) in 10 mM $MgCl_2$ supplemented with PACAP 1-38, PACAP 1-27 and PACAP 6-38 were incubated at 28 ° C for 38 h with continuous shaking at 160 rpm. Serial dilutions of the cultures were spread onto KB agar medium containing kanamycin and rifampicin, and the number of colonies was counted. Bars represent the mean ± SEM (n = 3 biologically independent experiments).

Supplementary Figure 4

A

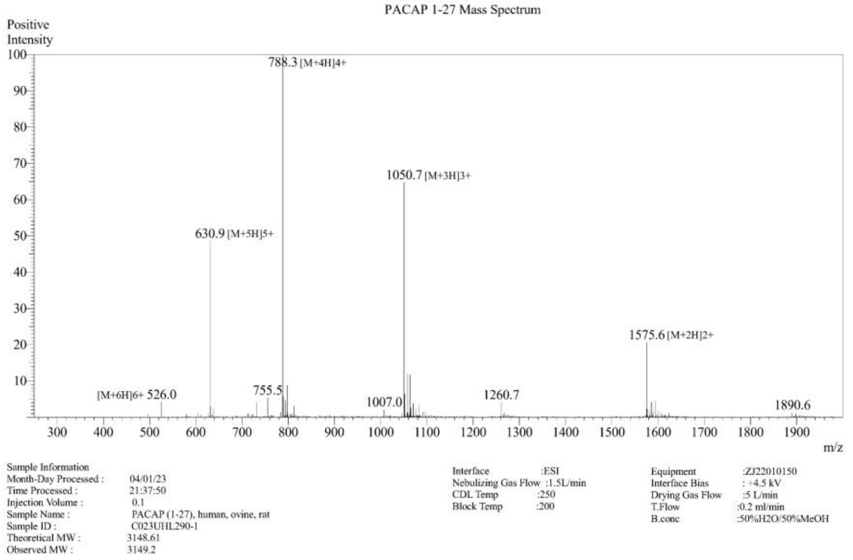

B

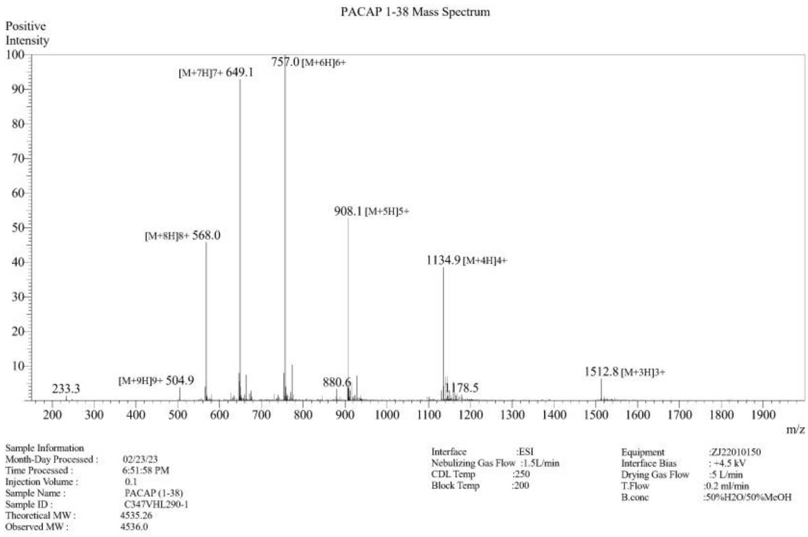

C

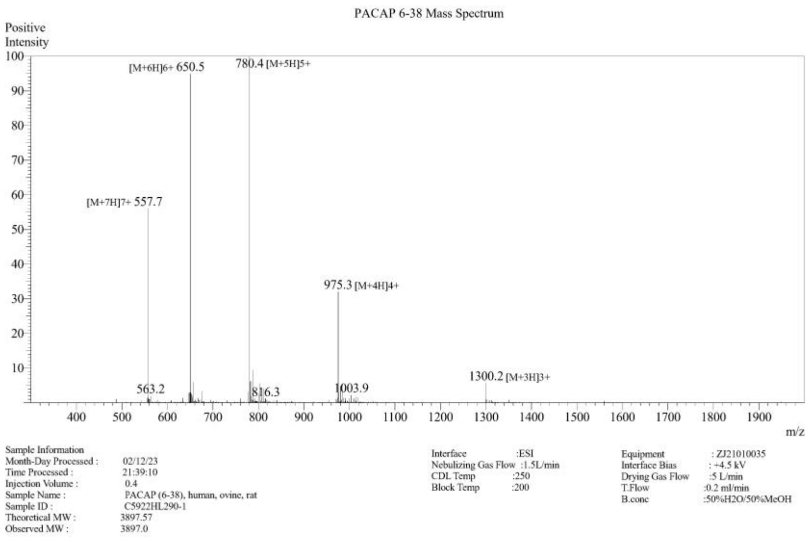

Supplementary Figure 4. Mass spectrometry (MS) analysis of synthetic peptides. Mass spectra of (A) PACAP 1–27, (B) PACAP 1–38, and (C) PACAP 6–38 are shown. The observed molecular ion peaks ([M+H]$^+$) correspond to the theoretical molecular weights of each peptide, confirming their successful synthesis and purity.

Supplemental Table 1. List of primers for plasmid construction.

pFRK1 F	CACCATCCCTGACAGTGAACTTCATTG
pFRK1 R	TTACTTAATTGAGCTGCTTTCTCTG

Supplemental Table 2. Primers used in the RT-qPCR.

AtFRK1-RT-qPCR-F	CGGTCAGATTTCAACAGTTGTC
AtFRK1-RT-qPCR-R	AATAGCAGGTTGGCCTGTAATC
AtWRKY29-RT-qPCR-F	ATCCAACGGATCAAGAGCTG
AtWRKY29-RT-qPCR-R	GCGTCCGACAACAGATTCT
EF1α-RT-qPCR-F	CAGGCTGATTGTGCTGTTCTTA
EF1α-RT-qPCR-R	GTTGTATCCGACCTTCTTCAGG
